# Data on air temperature, relative humidity and dew point in a boreal Sphagnum bog and an upland site (Shichengskoe mire system, North-Western Russia)

**DOI:** 10.1016/j.dib.2019.104156

**Published:** 2019-06-14

**Authors:** Dmitriy A. Philippov, Victoria V. Yurchenko

**Affiliations:** Papanin Institute for Biology of Inland Waters Russian Academy of Sciences, Russia

**Keywords:** Microclimate, Wetland, Lagg, Bog-island, Mire margin, Upland

## Abstract

The dataset contains microclimate parameters including air temperature, relative humidity and dew point measurements from a large wetland, Shichengskoe mire system, in Vologda Region, Russia, during four vegetation periods. Data were collected in 2013–2015 and 2017 using DT-171 data loggers (Elma Instruments). Data loggers were attached to the wooden posts at 0.5 m height from the surface. Continuous recordings were performed every 30 min providing 48 measurements of air temperature, relative humidity and dew point per day. The dataset presented in the article is of particular value to understanding the heterogeneity of abiotic parameters within mire systems.

**Table:** Specifications table

Subject area	*Physical geography*
More specific subject area	*Microclimatology*
Type of data	*Graphs*
How data was acquired	*DT-171 data loggers (Elma Instruments) with temperature and relative humidity sensors were used in the field to collect data.*
Data format	*Raw*
Experimental factors	*Data loggers were attached to the wooden posts at 0.5 m height from the surface, placed to the north side of the posts to avoid direct sunlight.*
Experimental features	*Measurements were conducted during the vegetation periods 2013*–*2015 and 2017. Continuous recordings were performed every* 30 min *providing 48 measurements of air temperature, relative humidity and dew point per day.*
Data source location	*Vologda Region, Russia, 59°56′37″N 41°17′10″E*
Data accessibility	*Raw data is archived in Mendeley Data*https://doi.org/10.17632/bpnd9978v6.1https://data.mendeley.com/datasets/bpnd9978v6/draft?a=526d6b54-d763-43ff-97a5-c4aa5c1e91f6
Related research article	*D.A. Philippov,* Specific features of structural organization of hydrobiocenoses in different-type of mire water bodies and water courses, Transactions of Papanin Institute for Biology of Inland Waters Russian Academy of Sciences, 79, 2017, 251–277 (in Russian) [1].

**Table:** 

**Value of the data** • The data is useful for investigating abiotic factors in wetland ecosystems. • The data is of particular value to the mire ecologists as it shows differences in microclimate parameters within a mire system. • These data can be useful for further studies of abiotic heterogeneity and edge effects in mire ecosystems. • The data can be beneficial for the research of associations between biotic components and local environmental factors (microclimate conditions) in mire ecosystems.

## 1 Data

**Fig. 1:**
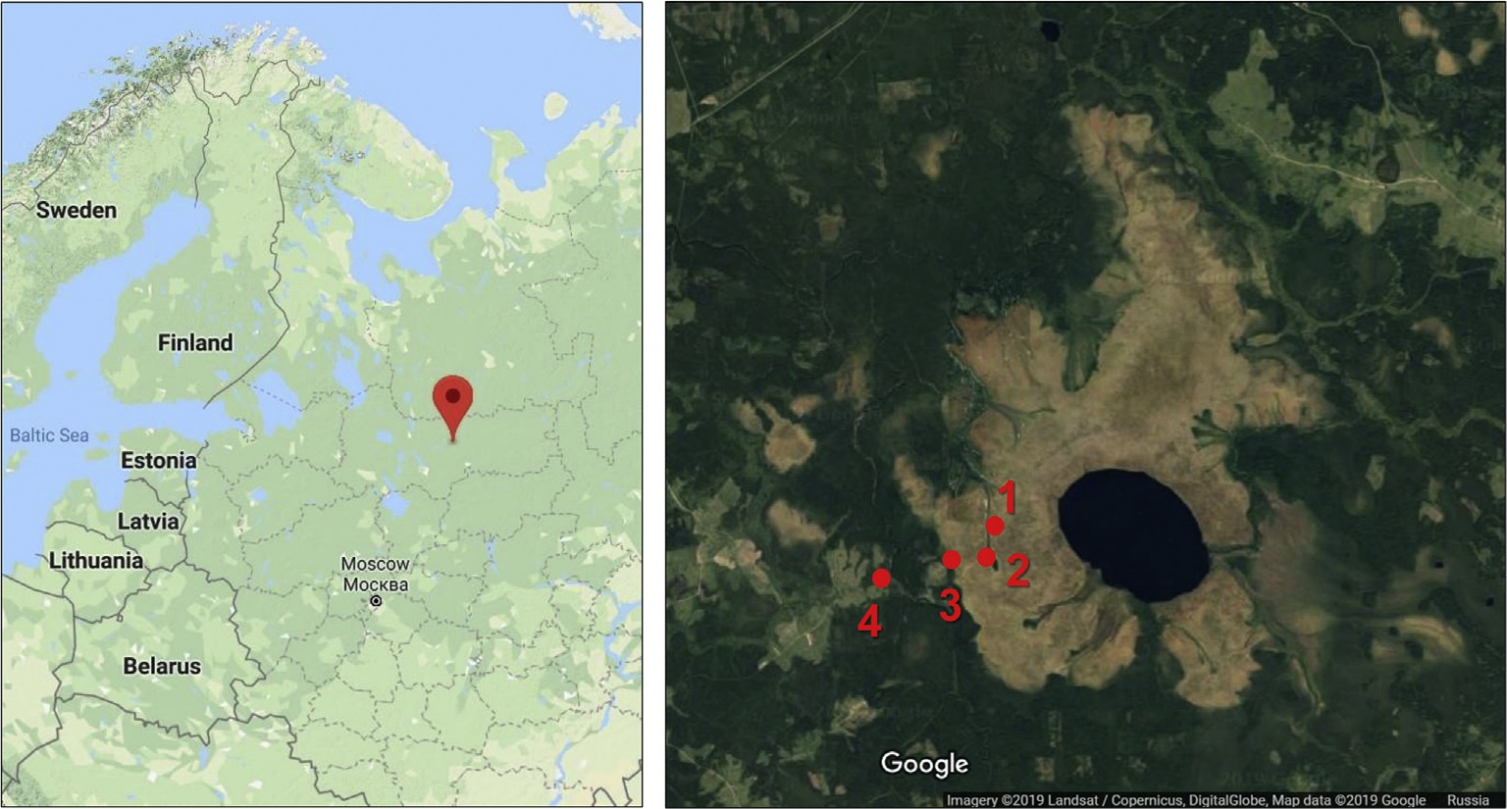
Data collection area (left) and data collection sites (right): 1 – lagg, 2 – paludified edge of a mineral bog-island, 3 – forested margin of the mire, 4 – upland.

**Fig. 2:**
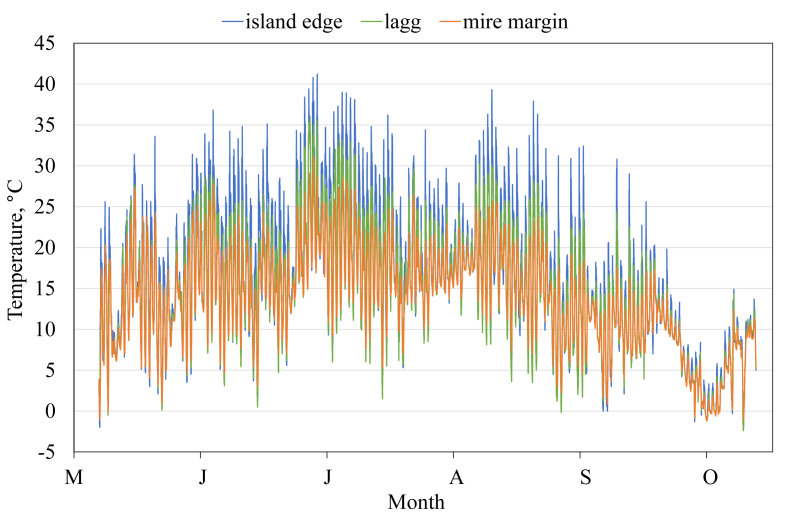
Air temperature during the vegetation period 2013. 1 – lagg, 2 – paludified edge of a mineral bog-island, 3 – forested margin of the mire.

**Fig. 3:**
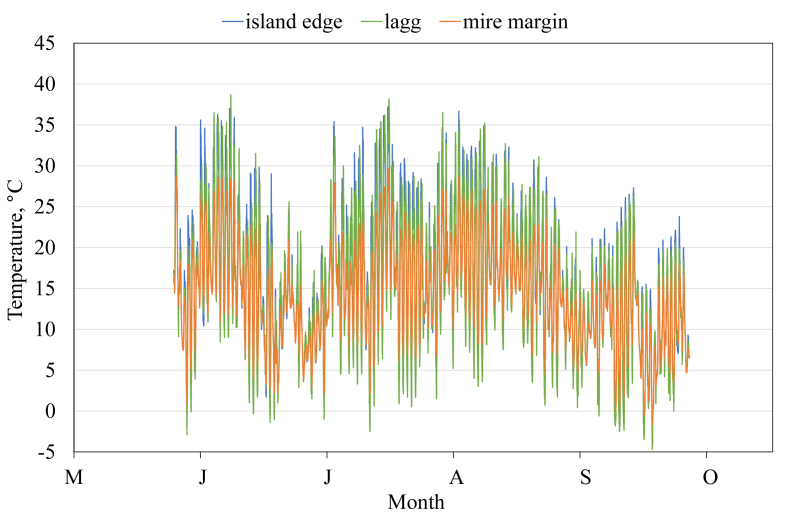
Air temperature during the vegetation period 2014. 1 – lagg, 2 – paludified edge of a mineral bog-island, 3 – forested margin of the mire.

**Fig. 4:**
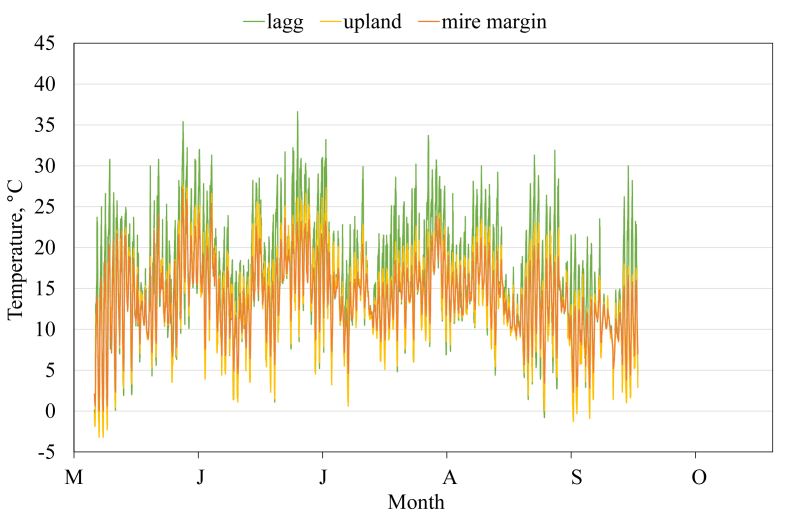
Air temperature during the vegetation period 2015. 1 – lagg, 3 – forested margin of the mire, 4 – upland.

**Fig. 5:**
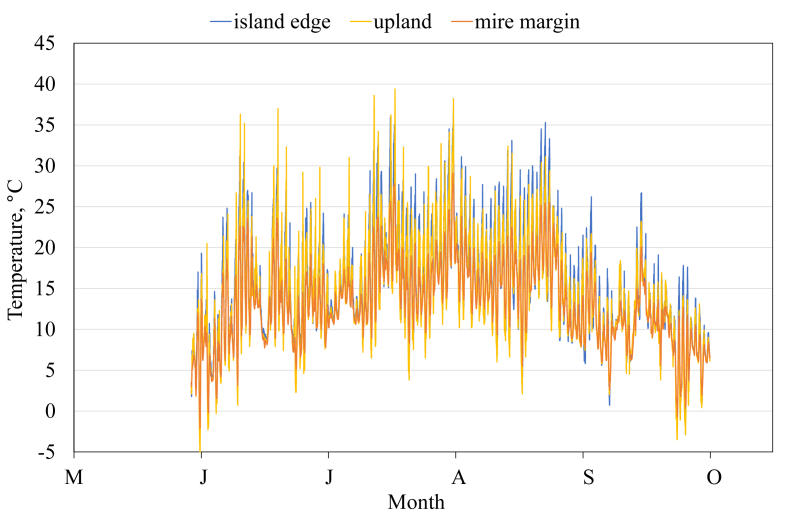
Air temperature during the vegetation period 2017. 2 – paludified edge of a mineral bog-island, 3 – forested margin of the mire, 4 – upland.

**Fig. 6:**
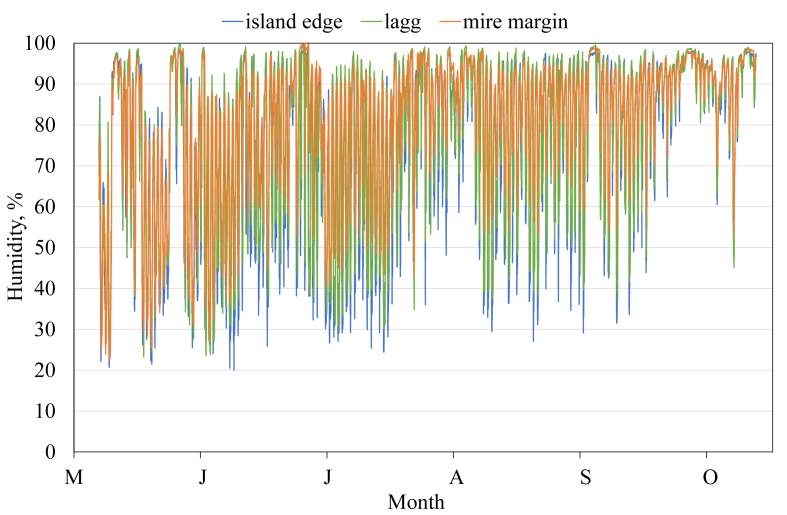
Relative humidity during the vegetation period 2013. 1 – lagg, 2 – paludified edge of a mineral bog-island, 3 – forested margin of the mire.

**Fig. 7:**
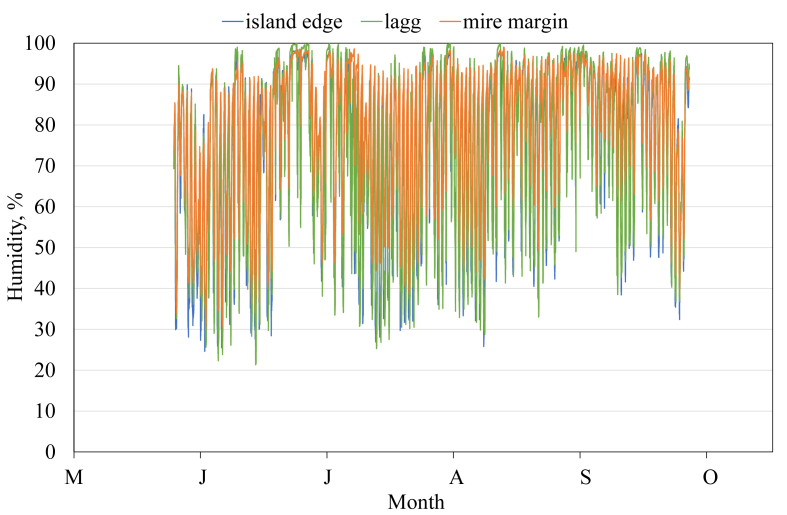
Relative humidity during the vegetation period 2014. 1 – lagg, 2 – paludified edge of a mineral bog-island, 3 – forested margin of the mire.

**Fig. 8:**
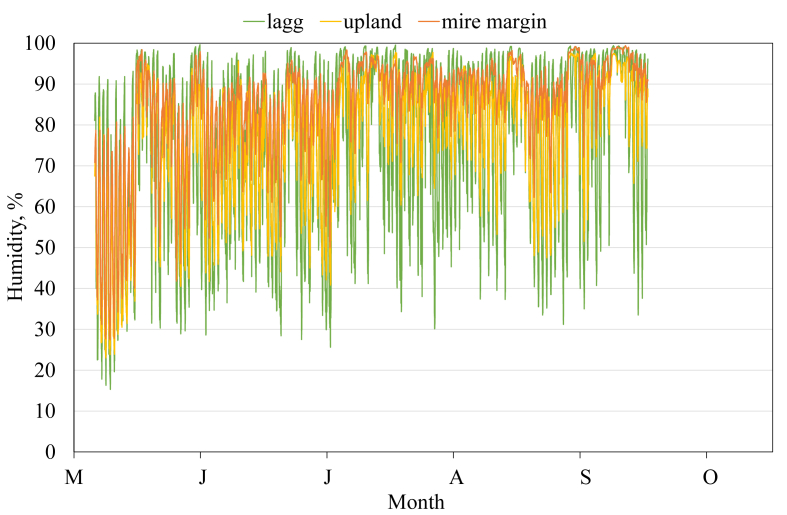
Relative humidity during the vegetation period 2015. 1 – lagg, 3 – forested margin of the mire, 4 – upland.

**Fig. 9:**
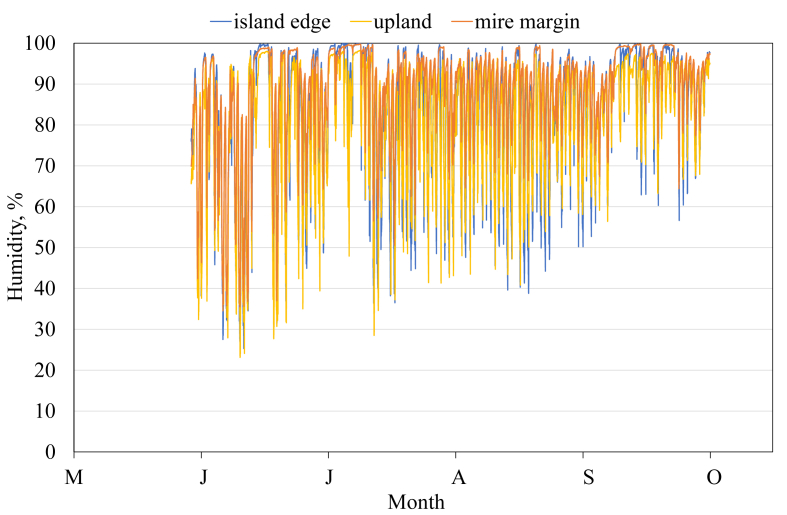
Relative humidity during the vegetation period 2017. 2 – paludified edge of a mineral bog-island, 3 – forested margin of the mire, 4 – upland.

**Fig. 10:**
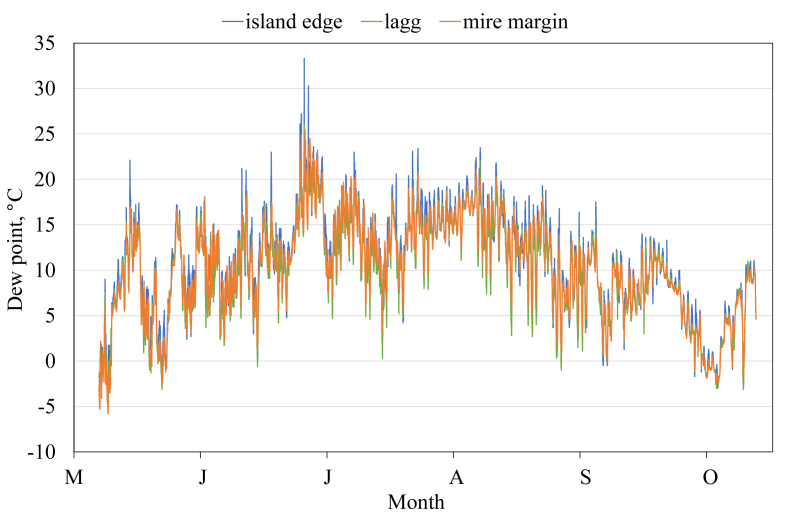
Dew point during the vegetation period 2013. 1 – lagg, 2 – paludified edge of a mineral bog-island, 3 – forested margin of the mire.

**Fig. 11:**
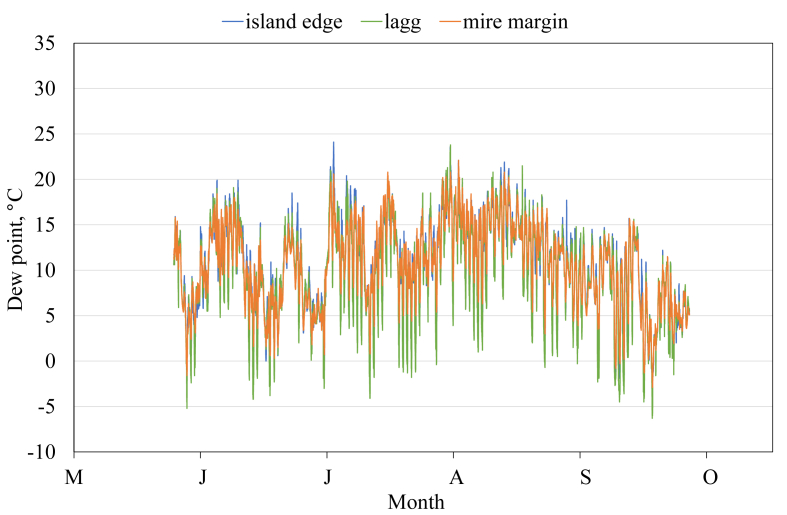
Dew point during the vegetation period 2014. 1 – lagg, 2 – paludified edge of a mineral bog-island, 3 – forested margin of the mire.

**Fig. 12:**
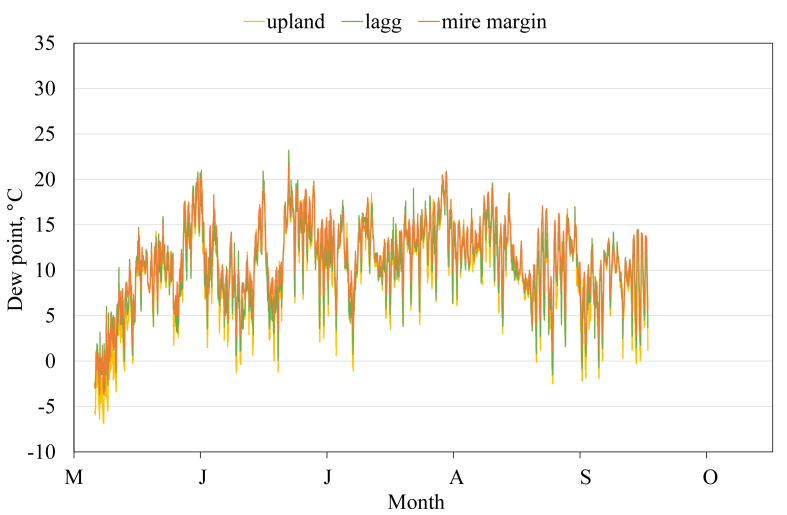
Dew point during the vegetation period 2015. 1 – lagg, 3 – forested margin of the mire, 4 – upland.

**Fig. 13:**
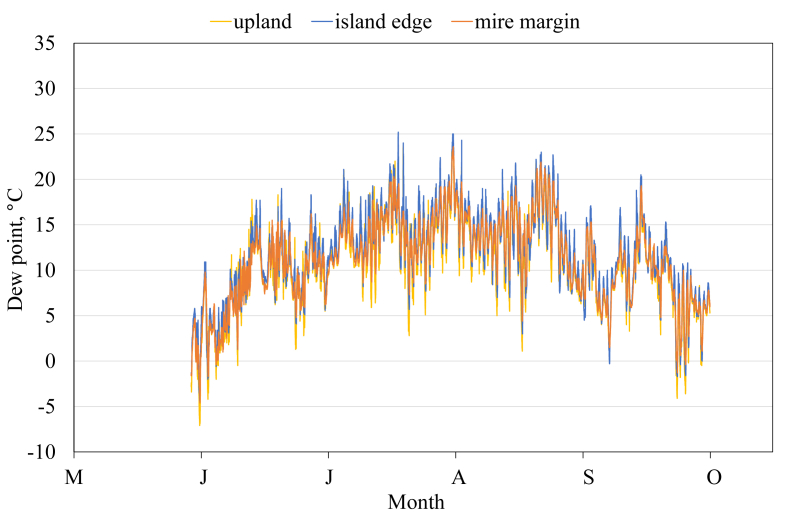
Dew point during the vegetation period 2017. 2 – paludified edge of a mineral bog-island, 3 – forested margin of the mire, 4 – upland.

This article presents spatial-temporal dynamics of microclimate within a large wetland, Shichengskoe mire system, in Vologda Region, Russia (Fig. 1), during four vegetation periods (2013–2015 and 2017). The microclimate parameters measured include air temperature, relative humidity and dew point. All parameters were measured in the field using DT-171 data loggers with temperature and relative humidity sensors. A total of 7584 measurements of each parameter were recorded in 2013, 5952 in 2014, 6384 in 2015, and 5952 in 2017. The dataset of raw data is available in Mendeley Data repository [2]. These data were used to construct graphs presented in this article (Fig. 2, Fig. 3, Fig. 4, Fig. 5, Fig. 6, Fig. 7, Fig. 8, Fig. 9, Fig. 10, Fig. 11, Fig. 12, Fig. 13) The data support and augment studies presented in the articles [1], [3].

## 2 Experimental design, materials, and methods

Data loggers DT-171 (Elma Instruments) with temperature and humidity sensors were used in the field. Overall temperature sensor working range is from −40°С to +70°С (with accuracy ±1 °C at the range from −10°С to 40°С and ±2 °C at the range from −40°С to −10°С and +40°С to +70°С). Overall humidity sensor working range is from 0 to 100% (with accuracy ±3% at the range from 40% to 60%, 3.5% at the range from 20% to 40% and from 60% to 80%, and 5% at the range from 0% to 20% and from 80% to 100%). Overall dew point working range is from −40°С to +70°С (with accuracy ±2 °C at 25 °C and relative humidity from 40% to 100%). No calibration procedure is required for the data loggers. All data loggers were set according to the user manual [4] and attached to the wooden posts at a height of 0.5 m from the surface at the northern side of the posts to avoid direct sunlight.

Four sites in total were chosen for data collection within Shichengskoe mire system (Fig. 1), namely: a lagg (59°57′01.0"N 41°17′08.5"E), a paludified edge of a mineral bog-island (59°56′36.5"N 41°17′10.1"E), a forested margin of the mire (59°56′19.7"N 41°16′06.9"E), and an upland (59°56′08.0"N 41°14′05.5"E). Continuous recordings (every 30 min) were performed for four vegetation periods, from 7 May to 11 October 2013 (158 days), from 25 May to 25 September 2014 (124 days), from 6 May to 15 September 2015 (133 days), and from 29 May to 29 September 2017 (124 days). Microsoft Office Excel software was used for the data storage and graph construction.
